# Clinical efficacy and safety of drug-eluting bead transarterial chemoembolization combined with targeted therapy and immune checkpoint inhibitors in the treatment of intermediate-to-advanced hepatocellular carcinoma

**DOI:** 10.12669/pjms.42.2.13021

**Published:** 2026-02

**Authors:** Hao Lu, Yuanlong Zhou, Chen Liu, Zan Li, Zhiyuan Luo

**Affiliations:** 1 Hao Lu Dept. of Hepatobiliary Surgery, Affiliated Hospital of Hebei University, Baoding 071000, Hebei, China; 2 Yuanlong Zhou Dept. of Hepatobiliary Surgery, Affiliated Hospital of Hebei University, Baoding 071000, Hebei, China; 3 Chen Liu Dept. of Hepatobiliary Surgery, Affiliated Hospital of Hebei University, Baoding 071000, Hebei, China; 4 Zan Li Dept. of Hepatobiliary Surgery, Affiliated Hospital of Hebei University, Baoding 071000, Hebei, China; 5 Zhiyuan Luo Dept. of Hepatobiliary Surgery, Affiliated Hospital of Hebei University, Baoding 071000, Hebei, China

**Keywords:** Adverse reaction, Drug-eluting bead transarterial chemoembolization, Intermediate-to-advanced hepatocellular carcinoma, Immune checkpoint inhibitor, Targeted therapy

## Abstract

**Objective:**

To investigate the clinical efficacy and safety of drug-eluting bead transarterial chemoembolization(DEB-TACE) combined with targeted therapy and immune checkpoint inhibitors(ICIs) in patients with intermediate-to-advanced hepatocellular carcinoma(HCC).

**Methodology:**

The clinical data of one hundred patients with intermediate-to-advanced HCC treated at The Affiliated Hospital of Hebei University between January 2021 to January 2024 were retrospectively analyzed. Patients were divided into the control group(n = 50) and the observation group(n = 50) according to the treatment regimen. The control group received DEB-TACE plus the targeted therapy lenvatinib, whereas the observation group was administered DEB-TACE plus lenvatinib and the ICI pembrolizumab. Clinical efficacy was assessed, adverse reactions occurring during the treatment period were recorded. Progression-free survival(PFS), overall survival(OS) and the cumulative survival rate (CSR) were compared between groups.

**Results:**

The observation group demonstrated significantly higher ORR and LCR compared with the control group (both *P* < 0.05). After treatment, serum CEA, CA199 and AFP levels were significantly reduced in both groups (all *P* < 0.05), with greater reductions observed in the observation group (*P* < 0.05). Post-treatment CD3^+^, CD8^+^ and CD4^+^ T-cell levels increased significantly in both groups (all *P* < 0.05), with higher levels in the observation group than in the control group(*P* < 0.05). The ARR did not differ significantly between groups (*P* > 0.05). No significant differences were observed in PFS or PFSR between groups(both *P* > 0.05).

**Conclusion:**

The combined use of DEB-TACE, targeted therapy and an ICI demonstrates superior clinical efficacy and a favorable safety profile, which can reduce tumor marker levels, enhance immune function and prolong OS in patients with intermediate-to-advanced HCC.

## INTRODUCTION

Treatment options for patients with intermediate-to-advanced hepatocellular carcinoma(HCC) remain relatively limited. Conventional chemotherapy can partially inhibit tumor growth; however, due to its non-specific cytotoxicity, it is often associated with severe systemic adverse effects, poor patient tolerance and suboptimal therapeutic outcomes.^1^ With ongoing advances in medical technology, interventional therapy has become an important treatment modality for intermediate-to-advanced HCC. Among these, drug-eluting bead transarterial chemoembolization (DEB-TACE) has attracted considerable attention for its unique advantages. This technique delivers chemotherapy drug-loaded microspheres directly to the tumor’s feeding arteries, enabling simultaneous vascular embolization to block nutrient supply and sustained local drug release.

This approach achieves higher intratumoral drug concentrations, enhances antitumor effects, minimizes damage to surrounding healthy tissue and reduces the incidence of systemic adverse reactions.^2^ Nevertheless, DEB-TACE alone has certain limitations in the treatment of intermediate-to-advanced HCC. Some patients experience tumor recurrence or metastasis after treatment, ultimately leading to treatment failure.^3^ In recent years, targeted therapy has made significant breakthroughs in HCC management. Targeted agents selectively act on specific molecular targets (*e.g*., vascular endothelial growth factor receptors, platelet-derived growth factor receptors) expressed on the surface or within tumor cells, thereby inhibiting tumor cell proliferation, invasion and metastasis and delaying disease progression.^4^ Concurrently, the advent of immunotherapy has brought new prospects for managing intermediate-to-advanced HCC.

Immune checkpoint inhibitors (ICIs), as a key class of immunotherapeutic agents, can reverse tumor-induced immune suppression, activate T lymphocytes and other immune effector cells and enhance the recognition and killing of tumor cells by the host immune system.^5^ To date, clinical studies investigating DEB-TACE in combination with targeted therapy and an ICI for intermediate-to-advanced HCC remain insufficient and the efficacy and safety of such treatment modalities require further validation. This study retrospectively analyzed one hundred patients with intermediate-to-advanced HCC and compared the clinical outcomes and adverse reaction rates (ARRs) of different treatment regimens, aiming to examine the clinical value of combining DEB-TACE with targeted therapy and an ICI and provide evidence-based guidance for the management of intermediate-to-advanced HCC.

## METHODOLOGY

The clinical data of one hundred patients with intermediate-to-advanced HCC treated at The Affiliated Hospital of Hebei University between January 2021 to January 2024 were retrospectively analyzed. Patients were assigned to either the control group(*n* = 50) or the observation group(*n* = 50) according to the treatment regimen. Baseline characteristics were comparable between groups, with no statistically significant differences(all P > 0.05), Table-I.

**Table-I:** Comparison of baseline characteristics between groups (*n*[%], [*χ̅*±*S*]).

Item	Control (n = 50)	Observation (n = 50)	χ^2^/t value	P-value
Sex			0.407	0.523
Male	35(70.00)	32(64.00)		
Female	15(30.00)	18(36.00)		
Age (years)	60.45±8.45	60.32±8.25	0.078	0.938
BCLC Staging Classification			0.044	0.834
Stage B	33(66.00)	32(64.00)		
Stage C	17(34.00)	18(36.00)		
Number of Tumor(s)			0.047	0.829
1	15(30.00)	16(32.00)		
≥2	35(70.00)	34(68.00)		
Maximum Tumor Diameter			0.178	0.673
<10 cm	18(36.00)	16(32.00)		
≥10 cm	32(64.00)	34(68.00)		
Child-Pugh Classification			0.396	0.529
Class A	34(68.00)	31(62.00)		
Class B	16(32.00)	19(38.00)		
Vascular Invasion			0.457	0.499
Absent	15(30.00)	12(24.00)		
Present	35(70.00)	38(76.00)		

### Ethical Approval:

The study was approved by the Institutional Ethics Committee of The Affiliated Hospital of Hebei University (No. HDFY-LL-2022-050; date: February 21, 2022) and written informed consent was obtained from all participants.

### Inclusion criteria:

- Diagnosis consistent with the criteria for intermediate-to-advanced HCC^6^, confirmed by both histopathology and imaging.
- Age ≥18 years.
- Complete clinical data, including general information, pre- and post-treatment imaging findings, laboratory test results and records of adverse reactions during treatment.
- Complete follow-up data.
- Provision of written informed consent before treatment, including agreement to receive the proposed treatment regimen and to allow the use of clinical data for research purposes.

### Exclusion criteria:

- Presence of other primary malignant tumors.
- Severe comorbidities, such as advanced cardiopulmonary dysfunction or coagulopathy.
- Failure to complete the planned combination therapy or change of treatment regimen during the course of treatment for any reason.
- Missing clinical or follow-up data.

### Treatment Methods:

Control Group: Patients in the control group received DEB-TACE in combination with targeted therapy:

### DEB-TACE Procedure:

Patients fasted from food and water for 6 hours before the procedure. Standard preoperative skin preparation was performed and venous access was established. Sedatives and antiemetic agents were administered 30 minutes before the procedure if necessary. Local anesthesia was achieved with 2% lidocaine. Using the Seldinger technique, the right femoral artery was punctured and an arterial sheath (typically 5-6 F) was inserted. A guidewire (*e.g*., hydrophilic guidewire) and an angiographic catheter (*e.g*., right heart catheter) were advanced through the sheath to the celiac trunk via the abdominal aorta (and to the common hepatic artery, if necessary).

Iodinated contrast medium (*e.g*., iohexol) was injected to perform digital subtraction angiography, identifying the tumor-feeding arteries (most commonly branches of the right or left hepatic artery), tumor staining intensity, the presence of arteriovenous fistulas or collateral circulation and excluding aberrant supply from normal hepatic tissue or gastrointestinal arteries. A microcatheter (*e.g*., 2.7F) was then exchanged and, under guidewire navigation, advanced superselectively into the primary tumor-feeding arterial branches, as close to the lesion as possible to minimize injury to normal hepatic tissue. After confirming accurate catheter positioning with a small contrast injection, the microsphere size was selected depending on tumor size and vascularity (commonly 100-700 μm, degradable or non-degradable).

The chemotherapy drug epirubicin was mixed with the microspheres, allowing the drug to be loaded onto or into the beads by adsorption (the drug-to-bead ratio was adjusted according to tumor burden). Under fluoroscopic monitoring, the drug-loaded beads were slowly infused via the microcatheter until substantial slowing or complete cessation of blood flow in the tumor-feeding artery (“stasis” endpoint) was achieved, avoiding reflux into normal vessels. Follow-up angiography was performed to confirm effective embolization (disappearance or marked reduction of tumor staining) and to ensure no non-target embolization of normal hepatic tissue. The microcatheter and angiographic catheter were withdrawn, the arterial sheath was removed and hemostasis at the puncture site was achieved by manual compression for 15-20 minutes, followed by elastic bandage compression dressing after confirming the absence of bleeding.

### Targeted Therapy:

Following resolution of post-embolization syndrome (typically 1-2 weeks after DEB-TACE), lenvatinib was initiated. Patients weighing ≥60 kg received 12 mg orally once daily and those weighing <60 kg received 8mg orally once daily, administered after breakfast. Observation Group: In addition to the regimen administered to the control group, patients in the observation group also received the ICI pembrolizumab at a dose of 200 mg per infusion, once every three weeks. Each cycle lasted 21 days and patients were treated for a total of four consecutive cycles.

### Outcome Measures:

Clinical Efficacy: Tumor response to the treatment regimens was evaluated according to the response evaluation criteria proposed in a previous study^7^, categorized as complete response (CR), partial response (PR), stable disease (SD), or progressive disease (PD). CR was defined as complete disappearance of target lesions; PR as a ≥30% reduction in the sum of the diameters of target lesions; SD as a reduction of <30% or an increase of <20%; and PD as a ≥20% increase in lesion size or the appearance of new lesions. The objective response rate (ORR) was calculated as: ORR = Number of CR cases + Number of PR cases / Total number of cases × 100% The local control rate (LCR) was calculated as: LCR = Number of CR + PR + SD cases / Total number of cases × 100%

### Tumor Markers and Immune Function:

Before and after treatment, 6 mL of fasting peripheral venous blood was collected. T-lymphocyte subsets in peripheral blood were determined by flow cytometry. And the above serum indexes were determined by enzyme-linked immunosorbent assay (ELISA). A portion of each sample was centrifuged to obtain serum for measurement of carcinoembryonic antigen (CEA), carbohydrate antigen 19-9 (CA199) and alpha-fetoprotein (AFP) levels using enzyme-linked immunosorbent assay kits (Shanghai Xuanzekang Biology Co., Ltd., Batch No. 101124, 120541 and 101221, respectively) in accordance with the manufacturer’s instructions. The remaining portion was analyzed by flow cytometry (NovoCyte Advanteon, Agilent Technologies [China] Co., Ltd.) to determine the levels of CD3^+^, CD8^+^ and CD4^+^ T cells.

### Adverse Reactions:

All adverse reactions occurring during the treatment period were recorded and compared between groups.

### Follow-up:

Patients were followed up after treatment via outpatient visits, inpatient reviews, or telephone contact. The follow-up endpoint was patient death or the cut-off date of June 2025. Progression-free survival (PFS) was defined as the time from the initiation of treatment to tumor progression or death from any cause. Overall survival (OS) was defined as the time from the initiation of treatment to death or the last follow-up date. PFS and OS were compared between groups.

### Statistical analysis:

All statistical analyses were performed using SPSS 26.0. Continuous variables were analyzed using the t-test, categorical variables were compared using the χ^2^ test and ordinal categorical data were analyzed using the rank-sum test. A *p*-value <0.05 was considered statistically significant.

## RESULTS

The ORR and LCR were significantly higher in the observation group than in the control group (both *P* < 0.05) Table-II. Before treatment, the serum levels of CEA, CA199 and AFP did not differ significantly between the two groups (all *P* > 0.05). After treatment, all three markers decreased significantly in both groups (all *P* < 0.05), with lower levels in the observation group than in the control group (*P* < 0.05) Table-III.

**Table-II:** Comparison of clinical efficacy between groups (*n*[%]).

Group	n	CR	PR	SD	PD	ORR	LCR
Control	50	2(4.00)	17(34.00)	17(34.00)	14(28.00)	19(38.00)	36(72.00)
Observation	50	4(8.00)	27(54.00)	14(28.00)	5(10.00)	31(62.00)	45(90.00)
Z/χ^2^ value		-2.667				5.790	5.263
P-value		0.008				0.016	0.022

**Table-III:** Comparison of tumor marker levels between groups (*χ̅*±*S*).

Group	n	CEA (ng/mL)		CA199 (U/mL)		AFP (ng/mL)	
		Pre-treatment	Post-treatment	Pre-treatment	Post-treatment	Pre-treatment	Post-treatment
Control	50	32.35±4.14	20.35±3.85*	54.23±6.48	30.54±5.86*	330.18±80.45	146.18±26.48*
Observation	50	32.14±4.25	16.84±3.42*	54.75±6.16	25.74±5.54*	331.02±82.19	106.24±24.82*
*t-value*		0.250	4.820	0.411	4.207	0.052	7.782
*P-value*		0.803	<0.001	0.682	<0.001	0.959	<0.001

Before treatment, no significant differences were observed between the two groups in CD3^+^, CD8^+^, or CD4^+^ T-cell levels (*P* > 0.05, respectively). After treatment, all three immune function parameters increased significantly in both groups (all *P* < 0.05), with higher post-treatment levels in the observation group compared with the control group (*P* < 0.05) Table-IV. The ARR did not differ significantly between groups (*P* > 0.05) Table-V.

**Table-IV:** Comparison of immune function markers between groups (%, *χ̅*±*S*).

Group	n	CD3^+^		CD8^+^		CD4^+^	
		Pre-treatment	Post-treatment	Pre-treatment	Post-treatment	Pre-treatment	Post-treatment
Control	50	36.45±6.52	40.35±5.23*	12.35±4.25	20.54±5.12*	28.24±5.42	34.75±4.58*
Observation	50	35.89±6.41	46.35±5.18*	12.24±4.16	27.72±5.26*	28.12±5.38	40.15±5.38*
*t-value*		0.433	5.764	0.131	6.917	0.111	5.404
*P-value*		0.666	<0.001	0.896	<0.001	0.912	<0.001

***Note:*** Compared with pre-treatment levels,

* P < 0.05 within the same group.

**Table-V:** Comparison of adverse reactions between groups (n[%]).

Group	n	Rash	Thrombocytopenia	Leukopenia	Hypothyroidism	Nausea and vomiting
Control	50	5(10.00)	6(12.00)	37(74.00)	1(2.00)	26(52.00)
Observation	50	4(8.00)	8(16.00)	44(88.00)	4(8.00)	28(56.00)
*χ^2^ value*		0.122	0.332	3.184	0.842	0.161
*P-value*		0.727	0.564	0.074	0.359	0.688

**Fig.1:**
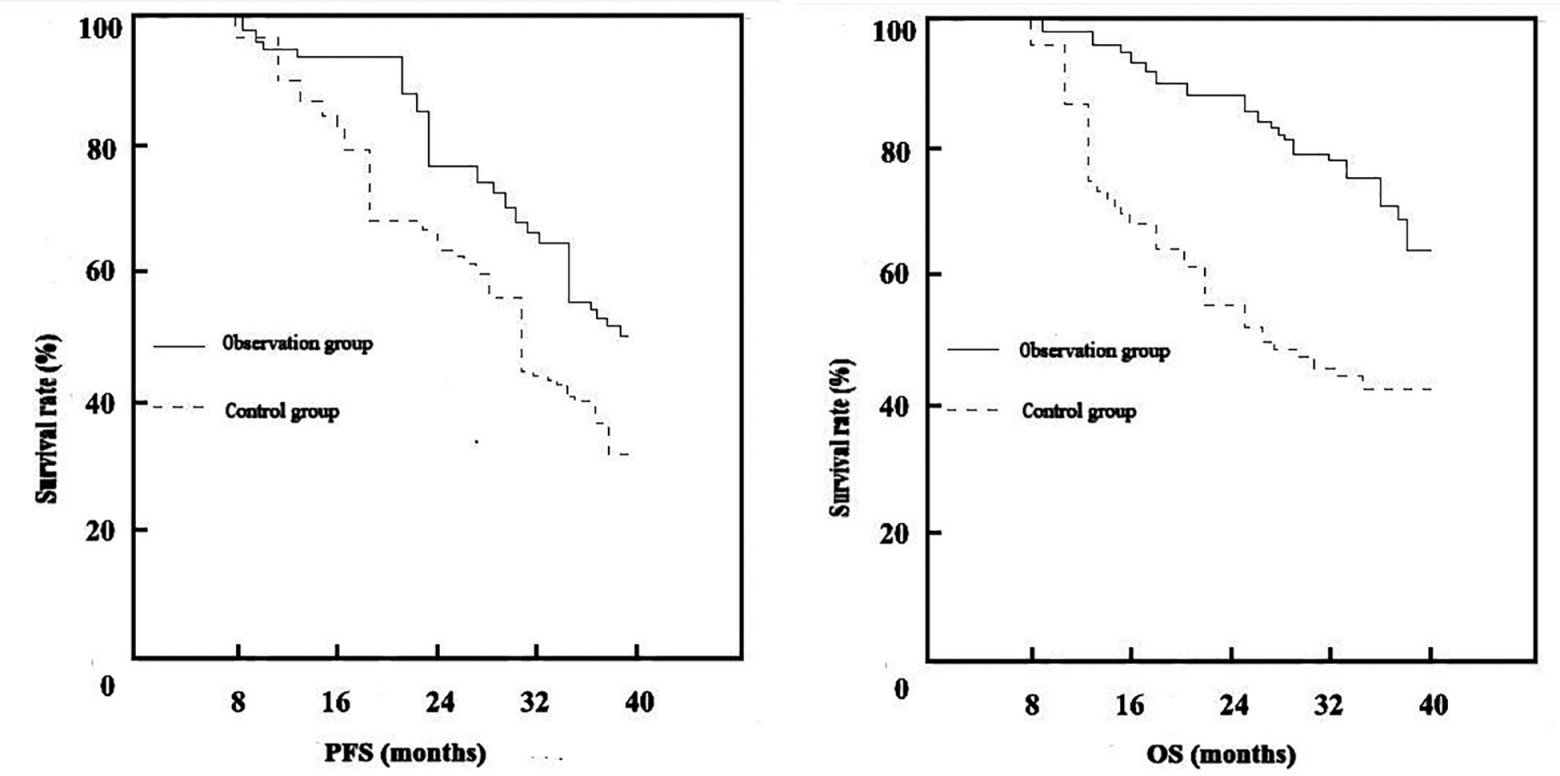
Comparison of PFS and OS between groups.

The median follow-up duration for all patients was 26.50 months. In the control group, the median PFS was 15.48 months, with a progression-free survival rate (PFSR) of 32.00% (15/50); median OS was 19.02 months, with a cumulative survival rate (CSR) of 44.00% (22/50). In the observation group, the median PFS was 18.90 months, with a PFSR of 50.00% (25/50); median OS was 25.70 months, with a CSR of 66.00% (33/50). No significant differences were observed in PFS or PFSR between groups (both *P* > 0.05). In contrast, the observation group had significantly longer OS and higher CSR compared with the control group (*P* < 0.05, respectively) Fig.1.

## DISCUSSION

This study retrospectively analyzed the clinical efficacy of DEB-TACE combined with targeted therapy and an ICI in patients with intermediate-to-advanced HCC. The results demonstrated that the observation group achieved superior outcomes in multiple parameters, providing valuable evidence for the treatment of intermediate-to-advanced HCC. From the perspective of clinical efficacy, both the ORR and LCR were significantly higher in the observation group than in the control group. This finding suggests that the addition of pembrolizumab to DEB-TACE plus lenvatinib can further enhance tumor control and treatment effectiveness. Mechanistically, DEB-TACE occludes the tumor-feeding arteries, thereby blocking nutrient supply while gradually releasing chemotherapeutic agents for direct cytotoxicity against tumor cells.^8^ Lenvatinib inhibits the VEGF/VEGFR signaling pathway, suppressing tumor angiogenesis, destabilizing existing tumor vasculature, triggering vascular leak and reducing tumor perfusion, thereby impeding tumor growth. Moreover, lenvatinib inhibits fibroblast growth factor receptors and blocks platelet-derived growth factor receptor alpha, thereby limiting tumor cell proliferation and reducing stromal cell-mediated nutritional support for tumor cells.^9^ The ICI pembrolizumab reverses tumor-induced immunosuppression by reactivating antitumor immune responses. The synergistic interaction results in enhanced antitumor efficacy.^10^ HCC is one of the most common malignant tumors worldwide, with persistently high incidence and mortality rates, posing a serious threat to human health.^11^ In China, the burden of HCC is particularly severe. Given its insidious onset and lack of specific symptoms in the early stages, most patients are diagnosed at the intermediate or advanced stage, thereby missing the optimal window for radical surgical intervention.^12^ Identifying effective treatment strategies for intermediate-to-advanced HCC has long been a focus and challenge in clinical research.

Regarding tumor markers, both groups showed significant post-treatment reductions in tumor marker levels, with the decrease being more pronounced in the observation group. CEA, CA199 and AFP are important markers for the diagnosis and monitoring of HCC and their reduction reflects a decrease in tumor burden.^13^,^14^ The significantly lower post-treatment tumor marker levels in the observation group further support the advantage of combination therapy in inhibiting tumor growth and reducing tumor activity, which is closely related to its more comprehensive antitumor effects. Changes in immune function markers are also noteworthy. Previous studies^15^ have shown that the complex tumor microenvironment in HCC can suppress immune function and promote immune evasion. CD3^+^, CD4^+^ and CD8^+^ T cells are critical components of the host immune system and their levels serve as important parameters for assessing immune status.^16^,^17^

In this study, the immune function markers of both groups significantly improved after treatment, with higher levels observed in the observation group. This suggests that the combination therapy can effectively enhance immune function. A possible explanation is that lenvatinib reduces the number of immunosuppressive cells, increases infiltration of effector immune cells, promotes immune cell migration and functionality and alleviates hypoxia, thereby strengthening antitumor immune responses.^18^ Meanwhile, pembrolizumab blocks the PD-1/PD-L1 pathway, restoring T-cell immune activity, enhancing the recognition and killing of tumor cells and ultimately improving immune function parameters.^19^

Furthermore, the combination of lenvatinib with an ICI can further alleviate the immunosuppressive state within the tumor microenvironment, thereby enhancing immune function. The more pronounced improvement in immune function markers observed in the observation group suggests that the addition of pembrolizumab resulted in more effective activation of the host antitumor immune response, which may be one of the key factors underlying the superior clinical outcomes in this group. In terms of safety, there was no significant difference in ARR between the two groups. This indicates that adding pembrolizumab to the treatment regimen maintains a favorable safety profile. Although ICIs may cause certain immune-related adverse reactions, in this study, appropriate monitoring and management effectively prevented a noticeable increase in ARRs, providing a degree of safety assurance for the clinical application of the combination therapy.

Regarding survival outcomes, the median OS and CSR were significantly higher in the observation group than in the control group, whereas no significant differences were observed in the median PFS or PFSR between the two groups. This suggests that the combination therapy may prolong OS but does not demonstrate a clear advantage in delaying disease progression compared with the control regimen. This might be explained by the onset time of action of ICIs, which typically produce more pronounced benefits in long-term tumor control, while their short-term impact on disease progression appears to be comparable to that of the control therapy.^20^ Nevertheless, the prolongation of OS is a critical measure of therapeutic efficacy and thus our finding holds important clinical significance.

### Limitations:

However, as this study is a retrospective analysis with a modest sample size and potentially insufficient follow-up, future large-scale, prospective, randomized controlled trials are warranted to further validate the efficacy and safety of this therapeutic approach.

## CONCLUSIONS

The combined use of DEB-TACE, targeted therapy and an ICI for the treatment of intermediate-to-advanced HCC can improve clinical efficacy, reduce tumor marker levels, enhance immune function, maintain a high level of safety, while also prolonging OS. These results support further promotion and application of this regimen in clinical practice.

### Authors’ Contributions:

**HL:** Conceived, designed. did statistical analysis & editing of manuscript, are responsible for integrity of research.

**YZ** and **CL:** Literature search, Did data collection and manuscript writing.

**ZL** and **ZL:** Did critical review..

All authors have read and approved the final manuscript. They are also account able for the integrity of the study.
